# Characteristics and factors influencing the volume of breastmilk donated by women to the first human milk bank in Vietnam

**DOI:** 10.3389/fgwh.2023.1185097

**Published:** 2023-10-02

**Authors:** Hoang Thi Tran, Tuan Thanh Nguyen, Oanh Thi Xuan Nguyen, Debbie Barnett, Gillian Weaver, Roger Mathisen

**Affiliations:** ^1^ Neonatal Unit and Human Milk Bank, Da Nang Hospital for Women and Children, Da Nang, Vietnam; ^2^ Department of Pediatrics, School of Medicine and Pharmacy, Da Nang University, Da Nang, Vietnam; ^3^ Alive & Thrive, FHI Solutions/FHI 360, Hanoi, Vietnam; ^4^ Milk Bank Scotland, Queen Elizabeth University Hospital, Glasgow, United Kingdom; ^5^ Human Milk Foundation, Harpenden, United Kingdom

**Keywords:** breastfeeding, breastmilk, donor human milk, human milk bank, newborn

## Abstract

**Background:**

Donor human milk (DHM) is essential to the operation of human milk banks (HMB). This study examined characteristics and factors associated with higher volumes of DHM donation at the first HMB in Vietnam.

**Method:**

Data from an online HMB monitoring system collected between February 2017 and July 2022 included demographic characteristics, child information, the timing of donation, and the volume of DHM. Higher volume is defined as equal to or greater than the median DHM volume per donor of 14.4 liters (L). Potential contributors to higher DHM volume were examined using the chi-square test in univariate and multivariable logistic regression analysis.

**Results:**

During the 5.5-year operation, this HMB recruited 517 donors with an average age of 28.6 years. Approximately 60.9% of donors had a college or higher degree and 97.3% gave birth in Da Nang city. Of these donors, the prevalence of cesarean birth was 48.2%, preterm births was 40.2%, and 27.9% had babies with a birth weight of less than 1,500 g. There was a similar proportion of donors between the hospital (48.2%) and community (51.8%). On average, hospital donors started their donations 15 days after birth when their newborns were 33.9 weeks corrected age compared to 63 days and 47.7 weeks among community-based donors. The overall median volume of DHM per donor was 14.4 L over a period of 46 days. The amount and duration were higher in community-based donors (17.5 L in 72 days, 300 ml/day) than those in the hospital (8.4 L in 30 days, 258 ml/day). More than 37% of donors contacted the HMB themselves; the remainder were introduced by health professionals. Factors associated with higher volumes of DHM included higher education (OR: 1.77; 95% CI: 1.09, 2.87), having a full-term newborn (OR: 2.46; 95% CI: 1.46, 4.13), and community-based donors (OR: 2.15; 95% CI: 1.22, 3.78).

**Conclusions:**

Mothers with higher education and from the community donate more breastmilk over a longer duration than those with lower education and from the hospital. Breastfeeding protection, promotion, and support should be offered to all mothers with specialized breastfeeding support for mothers of preterm and sick infants. This will ensure mothers have sufficient breastmilk for their newborns and potentially surplus breastmilk for donation.

## 1. Introduction

Breastfeeding is the biological norm for feeding infants and is vital for the survival and development of sick and very preterm newborns. When mothers' own breastmilk is not available, the World Health Organization (WHO) recommends donor human milk (DHM) from a human milk bank (HMB) as the next option to the mother's own breastmilk (1, 2). An HMB recruits and screens donors, stores, processes, screens and provides safe DHM to infants in need (3). Since the first HMB opened in Austria in 1909, there are almost 800 HMBs in 70 countries (3, 4). Although HMBs are operated differently across countries, the women who donate their surplus breastmilk are always essential to the operation of HMBs (5).

The first HMB in Vietnam was established in Da Nang in 2017 (6) with donors recruited from both the community and the Da Nang Hospital for Women and Children (DNHWC) where the HMB is located. During the last five years of operation, the HMB received more than 10,036 liters (L) of DHM and provided pasteurized DHM to 23,016 infants whose own mother's milk was not available or insufficient. Although this HMB is non-profit and receives valuable DHM for free, resources are needed for its operation and maintenance. Based on the expenditure on consumables, electricity, water, DHM transport, microbiological testing, garbage collection, property maintenance, depreciation, salary, and research and learning, the HMB estimated the price of pasteurized DHM and got approval from the Da Nang Department of Health. The current subsidized price of about USD 60 per L does not account for down payments of substantial startup investments and the level of efforts by the majority of staff involved are covered by other hospital departments such as initial donor recruitment, storing and distributing DHM as well as managing DHM refrigerators in the neonatal unit and postnatal wards (6, 7). Because social health insurance has not covered the cost of pasteurized DHM, families of vulnerable newborns must pay for its use. In addition to ongoing advocacy for social health insurance to cover the use of pasteurized DHM for vulnerable newborns, efforts to reduce and cover associated costs are needed.

An approach to reduce the operational cost is to focus on increasing donors who have the potential to provide a higher volume of DHM over a longer period. All donors in Da Nang are screened for human immunodeficiency virus (HIV), hepatitis B and C, and syphilis. DHM also needs to pass microbiological tests pre- and post-pasteurization. Because DHM is only pooled from one mother for pasteurization, the HMB must store individual DHM until a sufficient amount is available for pasteurization (6). These processes require resources with associated costs including the testing and electricity used. An operational research question for the HMB is how to identify potential women who could safely donate a higher volume of DHM for a longer duration. To address this knowledge gap, this study aims to investigate the characteristics of the donors and factors associated with a high volume of DHM using monitoring data from more than five years of operation of the first HMB in Vietnam. The study will also contribute to global data on human milk banking (8) and inform more effective donor recruitment and support strategies.

## 2. Methods

### 2.1. Study setting

Da Nang is a class-1 municipality city in the central coastal area of Vietnam with a total area of 1,284.73 square kilometers. It is the economic, cultural, and educational center of Central Vietnam. Economic revenue for Da Nang is from service (68.4%), industry and construction (20.4%), tax (9.2%), and agriculture (2.0%) (9). Da Nang has a population size of 1,195,490 and a crude birth rate of 18.68 per thousand population or approximately 22,400 infants born each year. The infant mortality rate in Da Nang City in 2021 was 8.19 deaths per thousand live births, which is lower than the national rate of 13.65 deaths per thousand live births (10).

DNHWC is a tertiary hospital for obstetrics, gynecology, and pediatrics, which has 1,200 beds and serves mainly three provinces with a population of 4.4 million in 2019–2020 (11). This hospital supports more than 15,000 births annually (11) and receives high-risk pregnancies and sick children as a referral hospital for the central region of Vietnam (11). The neonatal unit admitted approximately 4,000 newborns with more than 30% being preterm. The hospital is recognized nationally and internationally as a center of excellence for the implementation of Ten Steps to Successful Breastfeeding, early essential newborn care, and kangaroo mother care (KMC) (12, 13). The inclusion criteria for human milk donors include passing a questionnaire for medical history screening, general health status, and lifestyle (6). The questionnaire includes screening for medications that are contraindicated for breastfeeding, having received a blood transfusion, or tattoos within the previous six months (6). Donors must also pass the serological screening tests for HIV, hepatitis B virus, hepatitis C virus, and syphilis (6). Following completion of the screening process, accepted donors attend face-to-face educational training by HMB staff on hand hygiene, equipment cleaning, and DHM storage with final approval from the HMB manager to begin donation (6).

### 2.2. Study data

Data was collected during the normal operation of the HMB from February 2017 to July 2022. The data were collected and updated daily to a web-based package that captures all aspects of the donors including donor recruitment, screening, donor information, and volume of DHM (11). For this study, we extracted data directly from the HMB software and removed identifiable information from all records before data analysis.

### 2.3. Variables

An outcome variable for this study was the total volume of DHM, which was the summation of the amount of DHM of each donor. The donation duration was calculated by subtracting the donor approval date from the date of the last donation. The average volume of DHM per donor was calculated using the median volume of DHM per donor. For the regression analysis, we dichotomized the total volume using the median value: higher volume was defined as equal to or greater than the median volume of DHM per donor of 14.4 L.

The origin of the donor was defined at enrolment. A hospital donor begins donation while her newborn was cared for in the neonatal unit, while a community donor begins donation while her infant was at home. Thirty-seven out of 249 hospital donors continued donating breastmilk after their infants' discharge. We grouped them as hospital donors during the data analysis (intention-to-treat).

Maternal demographic characteristics including age, education, profession, and residency were recorded. Experience relating to the most recent birth was recorded including the number of children, place of birth, mode of birth, child sex, gestational age at birth, and birthweight.

In addition, we also extracted data on the sources of information relating to HMB and donation of breastmilk that the donors received and if the donor proactively approached HMB staff for donation.

### 2.4. Data analysis

We firstly performed descriptive analysis with stratification by donor origin for (1) demo- socio-economic characteristics and their birth experience, (2) sources of information relating to HMB and donation of breastmilk, and (3) volume and duration of donations. We presented descriptive statistics as the mean and standard deviation for normally distributed continuous variables or the median and interquartile range for skewed continuous variables. Counts and percentages were used for binary and categorical variables.

Secondly, we performed crude, binary analysis to examine the association between donors who contacted the HMB to donate, donor origin, donor characteristics, and birth experience for the prediction of a higher volume of DHM (≥14.4 L) using the chi-square test.

Thirdly, we performed adjusted logistic regression models with all the above covariates using Wald statistics.

The results were presented as crude and adjusted odds ratios with 95% confidence intervals. A two-tailed *p*-value of <0.05 defined statistical significance. We used Stata 15.1 (Stata Inc., TX, USA) to analyze the data.

## 3. Results

### 3.1. Donor characteristics

During five and a half years of operation, the HMB had 517 donors: 48.2% were recruited during inpatient stays, and the remaining 51.8% from the community (Table 1). The average age of the donors from both the hospital and the community was 28.6 years. Overall, 56.9% of the donors had a college higher degree of education and 60.9% had a white-collar job. Donors from the community had a higher education and job profile than those from the hospital (Table 1). Donors from the community were mostly living in Da Nang city (93.7%), which was higher than donors recruited from the hospital (45.8%) (Table 1).

**Table 1:** Characteristics of donors at the human milk bank from 2017 to 2022^a^.

	Hospital (*n* = 249)	Community (*n* = 268)	Total (*n* = 517)
Demo- socio-economic characteristics			
Age, y mean (SD)	28.2 ± 4.6	28.9 ± 3.7	28.6 ± 4.2
Education			
High school	100 (40.2)	33 (12.3)	133 (25.7)
Diploma	55 (22.1)	35 (13.1)	90 (17.4)
College, university and higher	94 (37.8)	200 (74.6)	294 (56.9)
Profession			
Housewives	63 (25.3)	34 (12.7)	97 (18.8)
White-collar jobs	115 (46.2)	200 (74.6)	315 (60.9)
Blue color jobs	41 (16.5)	17 (6.3)	58 (11.2)
Trader	30 (12.0)	17 (6.3)	47 (9.1)
Residence place			
Da Nang	114 (45.8)	251 (93.7)	365 (70.6)
Other provinces	135 (54.2)	17 (6.3)	152 (29.4)
Number of children			
1	140 (56.2)	172 (64.2)	312 (60.3)
2	93 (37.3)	87 (32.5)	180 (34.8)
3+	16 (6.4)	9 (3.4)	25 (4.8)
The most recent birth experience			
Sex			
Male	126 (50.6)	145 (54.1)	271 (52.4)
Female	123 (49.4)	123 (45.9)	246 (47.6)
Gestational age			
Mean (SD)	31.4 ± 3.9	37.7 ± 3.5	34.7 ± 4.9
<37 weeks	190 (76.3)	18 (6.7)	208 (40.2)
≥ 37 weeks	59 (23.7)	250 (93.3)	309 (59.8)
Birth weight, g mean (SD)	1,639 ± 736	3,022 ± 743	2,356 ± 1,012
Mode of births			
Vaginal	150 (60.2)	117 (43.7)	267 (51.6)
Cesarean	99 (39.8)	151 (56.3)	250 (48.4)
Place of birth			
DNHWC	233 (93.6)	175 (65.3)	408 (78.9)
Other hospitals in Da Nang	8 (3.2)	87 (32.5)	95 (18.4)
Hospitals in other provinces	8 (3.2)	6 (2.2)	14 (2.7)

DNHWC, Da Nang Hospital for Women and Children.

^a^ Data were presented as number (%) or mean ± standard deviation (SD) when specified.

### 3.2. Experiences relating to the most recent childbirth

Around 70.6% of the donors came from Da Nang City and 78.9% gave birth at the DNHWC where the HMB is located. The proportion of cesarean and vaginal births was similar among the donors (48.4% and 51.6%); 60.3% donated following the birth of their first child with 40.2% of donors having preterm infants, and 27.9% had babies with a birth weight of less than 1,500 g this time (Table 1).

### 3.3. Donor recruitment

The proportion of donors who proactively contacted the HMB was 37.1%, and this was higher in donors from the community (59.7%) than those from the hospital (12.9%) (Table 2).

**Table 2:** Sources of information relating to the human milk bank (HMB) and donation of breastmilk^a^.

	Hospital (*n* = 249)	Community (*n* = 268)	Total (*n* = 517)
Proactiveness in donation			
Health professionals contacted donors	217 (87.1)	108 (40.3)	325 (62.9)
Donors contacted HMB	32 (12.9)	160 (59.7)	192 (37.1)
People who talked with donors about donating human milk			
Neonatal unit staff	198 (79.5)	54 (20.1)	252 (48.7)
HMB staff	15 (6.0)	34 (12.7)	49 (9.5)
Postnatal staff	3 (1.2)	13 (4.9)	16 (3.1)
Antenatal clinic staff	6 (2.4)	7 (2.6)	13 (2.5)
Previous donors	0 (0.0)	8 (3.0)	8 (1.5)
Others	31 (12.4)	99 (36.9)	130 (25.1)
Other sources of information about donating human milk			
Facebook Fan page	20 (8.0)	142 (53.0)	162 (31.3)
Poster	20 (8.0)	22 (8.2)	42 (8.1)
Websites	11 (4.4)	19 (7.1)	30 (5.8)
Events	1 (0.4)	4 (1.5)	5 (1.0)
Newspaper, magazines	3 (1.2)	1 (0.4)	4 (0.8)
Brochures	1 (0.4)	0 (0.0)	1 (0.2)
Others	7 (2.8)	12 (4.5)	19 (3.7)

^a^ Data were presented as number (%) HMB, Human milk bank.

For donors from the hospital, the recruitment was done mostly by staff at the neonatal unit (79.5%), followed by HMB (6.0%), antenatal clinic (2.4%), and postnatal care (1.2%) (Table 2). Alternatively, the main sources of information for community donors were Facebook (53.0%), neonatal unit staff (20.1%), HMB staff (12.7%), posters (8.2%), and websites (7.1%). However, traditional mass media such as newspapers, magazines, and brochures were not a common source of information (Table 2).

### 3.4. Human milk donations

Throughout the duration of five and a half years, 517 women donated 10,104 L of DHM, of which 67.1% were from community donors (Table 3). On average, donors from the hospital started their donation 15 days after birth and when their newborns were 33.9 weeks of corrected age while donors from the community started 63 days after birth when their children were about 47.7 weeks. The average volume of DHM for each donor was 14.4 L over 46 days: the amount and duration were more than double in donors from the community than those in the hospital (17.5 vs. 8.4 L, 72 days vs. 30 days). The median daily volume of DHM was also higher among community donors as compared to hospital donors (300 ml vs. 258 ml) (Table 3).

**Table 3:** Volume and duration of donations to the human milk bank from 2017 to 2022.

	Hospital (*n* = 249)	Community (*n* = 268)	Total (*n* = 517)
Total DHM volume, L	3,322	6,782	10,104
Average donation duration, median (IQR) days	30.0 (20, 58.5)	72.0 (31.3, 118)	46.0 (24, 100)
Average volume of DHM, median (IQR) L	8.4 (5.4, 16.9)	17.5 (8.7, 26.3)	14.4 (7.6, 25.2)
Average volume of DHM per day, median (IQR) ml/day	258 (192–346)	300 (193–380)	279 (193–363)
Donation starting time after birth, median (IQR) days	15.0 (7.0, 34.0)	63.0 (26.0, 105.0)	31.0 (10.0, 75.5)
Child gestational age at starting of donation, mean (SD) weeks	35.1 ± 6.4	48.2 ± 9.8	41.9 ± 10.6
Child gestational age at finishing of donation, mean (SD) weeks	42.1 ± 8.8	60.8 ± 13.6	51.8 ± 14.9
Average donation duration, median (IQR) child days old	61.0 (37.0, 95.5)	149.0 (106.3, 202.5)	104.0 (54.5, 167)
Duration of donation of ≥46 days, *n* (%)	83 (33.3)	178 (66.4)	261 (50.5)
Volume of DHM ≥ 14.4 L, *n* (%)	81 (30.2)	178 (66.4)	259 (50.1)

DHM, Donor human milk; IQR, Interquartile range; SD, Standard deviation.

### 3.5. Associated factors for providing a high volume of DHM

In crude models (Table 4), a higher volume of DHM was positively associated with mothers' proactively approaching the HMB to become donors (OR: 2.15; 95% CI:1.50, 3.10), mothers with college, university, or higher education (OR: 2.28; 95% CI:1.60, 3.25), a white-collar job (OR: 1.49; 95% CI: 1.04, 2.12), full-term birth (OR: 4.05; 95% CI:2.81, 5.84), residency in Da Nang (OR: 2.33; 95% CI:1.57, 3.44), and being a community donor (OR: 4.10; 95% CI: 2.84, 5.92).

**Table 4:** Factors associated with a higher volume of pasteurized human donor milk.

	Donated at least 14.4 L		Crude model		Adjusted model	
	No (*n* = 258)	Yes (*n* = 259)	OR (95% CI)	*p*-value	aOR (95% CI)	*p*-value
Donors contacted HMB for donation, *n* (%)	73 (28.3)	119 (45.9)	2.15 (1.50, 3.10)	**<0**.**001**	0.82 (0.51, 1.32)	0.41
**Community donor, *n* (%)**	**90 (34.9)**	**178 (68.7)**	**4.10** (**2.84, 5.92)**	**<0**.**001**	**2.15** (**1.22, 3.78)**	**0**.**008**
Mother age (SD)	28.3 ± 4.5	28.8 ± 3.9	1.31 (0.92, 1.86)	0.14	1.45 (0.93, 2.25)	0.10
**College, university, or higher, *n* (%)**	**121 (46.9)**	**173 (66.8)**	**2.28** (**1.60, 3.25)**	**<0**.**001**	**1.77** (**1.09, 2.87)**	**0**.**02**
Obtained white-collar jobs, *n* (%)	145 (56.2)	170 (65.6)	1.49 (1.04, 2.12)	0.03	0.71 (0.44, 1.15)	0.71
Resided in Da Nang city, *n* (%)	160 (62)	205 (79.2)	2.33 (1.57, 3.44)	**<0**.**001**	0.95 (0.58, 1.55)	0.82
Had the first child, *n* (%)	155 (60.1)	158 (61.0)	1.04 (0.73, 1.48)	0.83	1.09 (0.70, 1.70)	0.72
Male, *n* (%)	133 (51.6)	137 (52.9)	1.06 (0.75, 1.49)	0.76	1.02 (0.70, 1.48)	0.94
**Full-term births, *n* (%)**	**94 (36.4)**	**181 (69.9)**	**4.05** (**2.81, 5.84)**	**<0**.**001**	**2.46** (**1.46, 4.13)**	**0**.**001**
Cesarean births, *n* (%)	120 (46.5)	130 (50.2)	1.16 (0.82, 1.64)	0.40	1.27 (0.86, 1.88)	0.23
Giving birth at DNHWC	224 (86.8)	184 (71.0)	0.37 (0.24, 0.58)	**<0**.**001**	0.66 (0.39, 1.12)	0.13

DNHWC, Da Nang Hospital for Women and Children. Adjusted odds ratios (aOR) and 95% CI from multiple logistic regression models, controlled for covariates in this table. The bold highlighted values corresponding to variables with statistically significant (*p* < 0.05).

In the adjusted logistic regression model (Table 4), factors associated with a higher volume of DHM included higher education (OR: 1.77; 95% CI: 1.09, 2.87, *p* = 0.02), having a full-term infant (OR: 2.46; 95% CI: 1.46, 4.13, *p* = 0.001), and community donors (OR: 2.15; 95% CI: 1.22, 3.78, *p* = 0.008).

## 4. Discussions

### 4.1. Hospital and community-based donors were mostly under 30 years of age

The demographic characteristics of women providing DHM to the first HMB in Vietnam at the DNHWC showed both similarities and differences when compared to other settings. The average donor age in this study was 28.6 years which is higher than the early- to mid-twenties found in Brazil and Indian studies (8) but similar to Chinese studies (14, 15) and younger than donors in Spain, Korea, Taiwan, or the US (8, 16, 17). Donors to the first HMB in Vietnam had higher education levels compared to donors from Brazil and similar to that from China, Norway, and Spain (8). Donors in this study had higher cesarean birth rates (48.4%) than those in China (32.8%) (15) or India (44.0%) (18). A potential explanation could be that this hospital is a referral facility that receives clients with high-risk pregnancies within the province and from other provinces in the central part of Vietnam (19). Cesarean births and vaginal births with episiotomy are barriers to breastfeeding (20–22). A study from eight countries in the Western Pacific region showed that where mothers received early and uninterrupted skin-to-skin contact in a supportive environment such as rooming-in practice and prohibiting formula use as policy, the breastfeeding rate was still high (23). Therefore, regardless of the mode of birth, early support and skin-to-skin contact could improve the likelihood of women establishing a good breastmilk supply and having surplus breastmilk for their infants and donations.

### 4.2. Mothers with higher education, from the community, and having full-term newborns donated more breastmilk

In this study, we found that donors with higher education, from the community, and having a full-term newborn were more likely to donate a higher volume to the HMB. Higher education was found to be associated with a higher volume of DHM. Previous studies have proved maternal higher education to be an important enabler for successful breastfeeding (24, 25). Mothers with higher education have more opportunities to seek information and are more confident in contacting HMBs directly for information or to donate. As found in a study from Brazil, higher education was a benefit for HMBs because more donors in this group donated more than one time (i.e., repeat donors) compared to the lower education group (26). Future support should focus on women with lower education by increasing their breastfeeding knowledge and skills to enable them to exclusively breastfeed their infants and, where appropriate, donate any surplus milk.

Mothers of full-term infants provided a higher volume of DHM compared with mothers of pre-term infants. There may be several reasons to explain this association. The mothers with preterm infants tended to donate human milk during their hospital stay and finishing after the infant was discharged. This can be seen by the infant age at the start of donation, 35.1 weeks, and the age at completion of the donation of 42.1 weeks. In contrast, mothers of healthy infants at home have greater comfort and the potential for a surplus of their own milk. In addition, support from family members was important to enhance breastfeeding (27, 28) and subsequently support human milk donation. These mothers expressed and stored milk for their infants for future use (e.g., after going back to work) and decided to donate some to the HMB. Studies from India showed that on average, hospital donors provided only 268 ± 386 ml (18). In comparison, a community donor from Taiwan could provide 17 L, and in Norway 28l (17, 29).

### 4.3. Although it is more challenging for donors from the hospital, their DHM is important

Staying in the hospital and caring for a preterm infant, can be challenging and a source of anxiety which may influence the mother's lactation and milk volume. In addition, the hospital's KMC rooms always have a high occupancy of mothers and newborns, sharing space and this would not always be optimal and comfortable for mothers to express breastmilk. However, KMC is an important intervention proven to prevent infant mortality and morbidity as strongly recommended by World Health Organization (1). Furthermore, mothers of preterm infants in our hospital roomed in with their infants 24 h a day for KMC which helped improve breastmilk supply thus supporting the donation of their surplus breastmilk. As donor milk from preterm mothers may have a higher protein concentration than that from full-term mothers, this provides more optimal nutrition for recipients who are preterm and sick (30) and so is a critical source of DHM for other preterm newborns. A systematic review of 28 studies of donor characteristics showed that preterm mothers accounted for a minority percentage, with only two studies from India and Brazil showing around half of the donors having preterm births (8). The large proportion of preterm donors in our setting is an important advantage for the HMB operation and reflects good practice for preterm mothers in neonatal units by maintaining exclusive human milk feeding when their infants are still premature and not able to have full direct breastfeeding.

There are fewer donors from hospitals with less volume of DHM in many HMBs worldwide (8). Conversely, hospital donors who often have preterm infants staying in the neonatal unit, are accessible for conversations about donation as well as transporting DHM within the hospital. Therefore, improving recruitment within this group is beneficial to the HMB. To support these women and to promote their donation of breastmilk whilst resident in the hospital, reasonable measures should be made to improve facilities including additional comfortable spaces and bathrooms, and support mothers with preterm infants to begin early breastmilk expression even when their newborns are in the neonatal intensive care unit. It is important to provide coaching on the hand-expression of breastmilk, knowledge on handling of expressed breastmilk, and suitable facilities for breastmilk storage for their infants. Throughout, the act of donation of breastmilk to an HMB should never be a disadvantage to the donor or her infant.

Most hospital donors started donating DHM when their infant was around one month. The age of the infants of donors from the hospital was much lower than community-based donors (15 vs. 63 days). Overall, this was still lower than those from Poland where the donors started their donation around 14 weeks after birth (31), and Norway at 7 weeks. Similarly, a study from Taiwan showed less than 16% of the first donation happened in the first 2 months. The systematic review of donor characteristics also showed most donors provided DHM after their infant was one month old (8). The earlier donation seen in our HMB may have resulted from the fact that mothers were encouraged to practice early, continuing KMC, and received specialized breastfeeding support in the neonatal unit. Globally, community donors are usually encouraged to wait until their breastfeeding is fully established before starting to express breastmilk for donation. This is to help prevent the overproduction of breastmilk to the detriment of the donor.

Although the time to start the donation for hospital donors was earlier than for community donors the duration was shorter. More than 54% of hospital donors came from other provinces so their donation period would end once the infants were discharged. The duration of the donation was shorter in this first HMB in Vietnam as compared to HMBs in Norway or Korea (about one month vs. more than two months) (16, 29). Future interventions should focus on improving the duration of the donations, if appropriate and not likely to disadvantage the mother or her child. Enabling more optimal conditions for lactation and breastfeeding could encourage earlier donations for both hospitals and, where appropriate, community donors. In Vietnam, maternity leave is six months for women working in formal sectors. If the donation period started within two months following births, as in the community donors in this study, then the donation time potentially could be maximized for up to four months. The HMB should consider investing more in encouraging mothers in the community to donate to maximize the potential of a longer duration of donation if ethically and practically appropriate.

### 4.4. The average volume of DHM and comparison with other HMBs in the world

The median volume of DHM from donors in this study was much higher than that from India (18) and relatively higher than a report from the North American HMB with a median of 11.4 L (32). However, the volume of donors in Da Nang was much lower than that from Taiwan or Norway with a median of 17 L and more than 28 L respectively (17, 29). A shorter donation period and lower volume compared to several HMBs may be influenced by the hospital donors where their duration and volume were much lower than that of the community. In addition, the median daily volume from hospital donors was also lower than that from community donors (258 ml vs. 300 ml). In DNHWC, breastfeeding support policies were implemented from antenatal consultations, then early and continuing skin-to-skin contact was provided to all preterm newborns who did not require resuscitation. In the neonatal unit, all preterm newborns were provided continuing KMC by the mother and family members. Neonatal staff promoted and supported early breastmilk expression. Commercial milk formula (CMF), feeding bottles, and pacifiers were prohibited. Pasteurized DHM was provided if the mother's own milk was not sufficient. Each KMC room had a refrigerator with a freezer compartment for mothers to store breastmilk for their infants. For infants who were not able to breastfeed themselves and required alternative methods of the gastric tube or cup feeding, mothers express breastmilk at least eight times a day. While very small newborns in the neonatal unit only required small amounts of breastmilk, these mothers would have a surplus to donate to the HMB after reserving what was needed for their infants. Supporting donors with parent facilities, a comfortable space, individual breast pumps, and sterilizers or coaching on hand expression based on informed decisions would promote further human milk feeding and donation in the neonatal unit (33).

### 4.5. The importance of health staff and social media in donor recruitment strategies

Finally, to encourage donor recruitment, we have used various measures to approach potential donors including health professional consultation, fan pages, posters, and other written materials. About one-third of donors contacted our HMB by themselves while most donors were referred by health staff. These were often hospital donors whom health professionals recognized their potential and HMB staff visited them to encourage recruitment.

Health professionals were a major source of communication and information sharing for potential donors in Da Nang. This was also reported in a Brazilian study (26). On the contrary, online sources played important roles in China and Korea (8). As around half of our donors came from the hospital, the role of health professionals is important, especially in the Neonatal unit where mothers of newborns in KMC remained with their infants. This supports the integration of breastfeeding support and donor recruitment by neonatal staff.

Facebook is the second most common measure in providing information on donations to the HMB. In Vietnam, as well as other countries, the internet is widely used, especially by women between the age of 20–30 s who grew up with access to the internet. Social media is now commonly used by mothers (34), for information on breastfeeding, HMB, and human milk donation. There are more articles written and available on public media and social network sites rather than on traditional media such as newspapers or magazines. A Chinese study in 2013–2016 on 2,680 donors from 14 HMBs showed that most of the information came from the internet (32.5%), then health professionals (29.4%), television (14.9%), newspapers, and magazines (10.1%) (15). In our study, apart from Facebook, other methods were not popular. Advertising HMB on television and in newspapers often requires a fee although free advertising was given on a few occasions. Therefore, free social networks would be preferable. The HMB should invest in increasing information and confidence in breastfeeding and raising awareness and encouraging donations via social media. Other measures should be enhanced including education on donation and donor recruitment for health professionals from the public health system where infant immunization occurs and encouraging television and newspapers to participate in breastfeeding promotion and donation whenever appropriate.

### 4.6. Strengths and limitations

To our knowledge, this study is among the few studies that have examined characteristics of donors and the volume of DHM donated to an HMB in lower-middle-income countries. The use of the online system with structured forms and pre-coded options as well as built-in verification functions helps to reduce the workload, cost, and reduced recall bias while ensuring a large sample size and the quality of the data. In addition, because the data from this monitoring system are regularly used to optimize the functionality of the HMB, it ensures compliance with standardized protocols, facilitate networking and information sharing among HMBs, and that the quality of the data is verified and improved.

Our study also has limitations. First, the intensity of recruitment of donors might vary depending on the demand of DHM. Secondly, the characteristics of donors might have been different during the COVID-19 pandemic in Da Nang (from March 2020 to July 2022), when the number of recipients decreased because there was a decrease in referrals of high-risk pregnant women and newborns from surrounding provinces but also fewer donors and DHM due to the lockdown (11, 35). However, the coverage of five and a half years would still capture key characteristics of donors. It is beyond the scope of this study, but this study could have benefitted from some qualitative data, which would explain how potential donors are reached as well as barriers and motivators for donation.

## 5. Conclusions

Women enabled to breastfeed their infants according to the WHO recommendations and willing to donate their surplus breastmilk is essential for the sustainable operations of HMBs. Mothers with higher education levels from the community donate more breastmilk over a longer duration compared with those with lower education levels and from the hospital.

We recommend that HMBs focus on community-based donors with higher education levels by providing information about HMB and breastmilk donation. This subsequently can stabilize operational expenses at the HMB, which is of paramount importance when the costs for the use of HDM are ultimately recovered by charging these vulnerable families and with the limited public financing mechanisms currently available. Donor engagement and support strategies for the HMB should continue focusing on universal breastfeeding protection, promotion, and support to enable and sustain donations of surplus breastmilk. Health workers are to provide targeted and specialized support based on care needs and risk assessments of more vulnerable mother-and-infant dyads when these mothers show a willingness to provide their surplus breastmilk while at the hospital. Also, health workers would need to provide tailored support to donors from the hospital so that they continue donation after the hospital discharge.

## Data Availability

The original contributions presented in the study are included in the article, further inquiries can be directed to the corresponding author.
